# Secular trends in body height, body weight, BMI and fat percentage in Polish university students in a period of 50 years

**DOI:** 10.1371/journal.pone.0220514

**Published:** 2019-08-01

**Authors:** Ewa Kalka, Anna Pastuszak, Krzysztof Buśko

**Affiliations:** 1 Clinic for Children and Youth, Institute of Mother and Child, Warsaw, Poland; 2 Team Projects, Institute of Sport–National Research Institute, Warszawa, Poland; 3 Department of Anatomy and Biomechanics, Kazimierz Wielki University, Bydgoszcz, Poland; McMaster University, CANADA

## Abstract

**Objectives:**

The aim of the study was to determine changes in the magnitude and direction of secular trends in body height, body weight, body mass index (BMI) and fat percentage in university students from a university of technology and a university of physical education in a period of 50 years.

**Methods:**

The data were derived from the examinations of male students from the Warsaw University of Technology, conducted four times, in 1959, 1971, 1994, 2011, and male students from the Józef Piłsudski University of Physical Education in Warsaw, who were examined in 1963, 1972, 1996, and 2012. Body height, body weight and thickness of 2 skinfolds (triceps skinfold and abdomen skinfold) were measured. Body mass index (BMI) and fat percentage (FAT%) were also calculated.

**Results:**

Current university students are taller and heavier than their peers from the previous decades, with BMI remaining within the reference range. A substantial increase in fat percentage was found in both groups. Over the period of fifty years, mean fat percentage in students from the university of technology increased by 6.3% (F1,3 = 116.56, p < 0.001, η^2^ = 0.3736), whereas this increase in the students from the university of physical education rose by 3.5% (F1,3 = 72.94, p < 0.001, η^2^ = 0.3181).

**Conclusion:**

Changes in secular trends in the students from both universities are likely to be linked to the dynamic economic and systematic transformation in Poland observed in the period of the last 50 years. The period of economic transformations in the last decade was more conducive to physical development of university students than the previous period of economic crises.

## Introduction

The problems of intergenerational changes in body size concerning secular trends in somatic characteristics and acceleration of human biological development are among the most important research problems in the field of anthropology. Previous data in the literature provided the evidence that tendencies for changes in somatic characteristics are noticeable both in Poland and all over the world [1–2].

Examinations of the biological status of successive generations in the period of social, economic and urbanization transformations show their varied effect on the development of morphological characteristics of the population. On the one hand, indices of physical development show upward tendencies, reflected by the increasing body height and body mass in successive generations of young people. On the other hand, some unfavourable tendencies have been documented for the worrying percentage of people with obesity and overweight. This phenomenon is widespread in Europe and worldwide [3–4].

Numerous studies published in recent years have documented several-decade analyses of secular trends in various populations, ranging from early years of the last century to the present [5–6]. The findings of these studies lead to the conclusion that the secular trends in many European countries and all over the world continue to be observed, and, undoubtedly, will be further examined in the decades to come and used to prepare and update the developmental standards of body dimensions in the populations. The data on body size in a specific population from the last century are valuable only from the historical and comparative standpoint.

In Poland, the phenomenon of secular trends of the adult population has been analysed most often based on the results of examinations of military recruits and university students, including students from the Warsaw University of Technology (WUT) and Józef Piłsudski University of Physical Education in Warsaw (UPE). These examinations have been repeated since the 1950s [7–8]. The secular trend in body height of Polish military recruits ranged from 0.8 to 2.4 cm per decade [7]. Similar dynamics of changes was observed in most European countries, with body height increasing in the last 150 years from 1 to 3 cm per decade [9].

With respect to the students from WUT, body height increased in a period of 35 years (1959–1994) by 6.6 cm [8]. A steady increase in body fat percentage and body circumferences was also observed. Furthermore, comparison of typological components of body build of the students from UPE using the Heath-Carter concept showed a significant rise in endomorphy, with mesomorphy and ectomorphy components remaining unchanged in a period of 24 years (1972–1994). The need to verify whether the phenomenon of the secular trend continues to be present in the above universities inspired further anthropological research in 2011–2012.

The aim of the study was to determine changes in the magnitude and direction of secular trends in body height, body mass, body mass index (BMI) and fat percentage in university students from a university of technology and a university of physical education in a period of 50 years.

## Methods

### Ethics statement

Ethical approval for the study in 2011 and 2012 was provided by the Senate Research Ethics Committee of the Józef Piłsudski University of Physical Education in Warsaw, Poland (SKE 001-82-1/2010) and conducted according to the Declaration of Helsinki. Participants were informed (both in writing and orally) about all testing procedures. All participants gave their written consent to participate in the study. Furthermore, they were aware of the possibility to withdraw consent at any time for any reason.

#### Participants

The examinations were conducted in two groups of university students. The first group comprised first or second year male students from the Warsaw University of Technology (WUT), who were not involved in either professional or amateur sports (WUT, n = 589), examined in 1959, 1971, 1984, 1994 and 2011 using the same research procedure in each examination. The research group was randomized among students from all the faculties according to the same rule: random selection of names from the list of students in individual faculties, number of study participants from a specific faculty proportional to the fraction of the students from this faculty in the total number of students in a specific year of studies; study participants were not involved in professional sports; all subjects had Polish nationality and were Caucasians.

Anthropometric measurements were conducted always between November and December.

The second group of men studied were students from the University of Physical Education in Warsaw (UPE, n = 473). The measurements were conducted four times, in 1963, 1972, 1996, and 2012. All the first or second year students from the Faculty of Physical Education were measured. All the male students had Polish nationality and were Caucasians. The examinations were always scheduled for the period between October and November.

#### Anthropometric measurements

Body mass, body height and 2 skinfolds (triceps skinfold and abdomen skinfold) of all students (from both universities) were measured using a set of measurement instruments (SiberHegner, Switzerland). All the measurements were performed by anthropologists in accordance with the procedures described by Martin and Saller [10]. Body height, body weight and thickness of skinfolds were evaluated with accuracy of 1.0 cm, 0.1 kg and 1.0 mm, respectively. During the measurements, students were barefoot and wearing light underwear.

The somatic characterization of students was prepared based on body weight, body height, body mass index (BMI) and body fat percentage. The BMI is defined as the body mass divided by the square) of the body height, and is universally expressed in units of kg/m^2^, resulting from mass in kilograms and height in metres. Body fat percentage (FAT%) was calculated using the method proposed by Piechaczek [11].

The following equation was used for computation of body density in men:

D = 1.125180–0.000176 x log x_1_−0.000185 x log x_2_

Where:

D = body density,

x_1_ = triceps skinfold,

x_2_ = abdomen skinfold.

Estimation of fat percentage was based on the Keys and Brožek equation [12].

FAT% = 100 x ((4.201 x D^-1^)– 3.813)

Where:

FAT% = percentage of adipose tissue,

D = body density.

Details of the method are contained in a study by Piechaczek [11]. Piechaczek's equation has been regularly used for evaluation of body tissue composition (content of fatty tissue and active tissue) in students from both universities in all examinations.

### Statistical analysis

Comparison of the results between the groups of students from the same university in different years and between universities was based on the univariate analysis of variance (ANOVA). Significance of differences was verified by means of the post hoc Scheffé test. Mean difference and 95% confidence intervals (CI) were calculated if the post hoc procedure was necessary. In order to interpret the effect size for statistical differences in the ANOVA we used eta square classified as small (0.01<η^2^≤0.06), medium (0.06<η^2^≤0.14) and large (η^2^>0.14).

Multiple regression analysis was employed to predict mean values of the analysed characteristics for entire decades. For WUT, these decades were 1969, 1979, 1989, 1999, 2009, whereas for UPE they were 1973, 1983, 1993, 2003 and 2013.

Secular trend analyses were conducted for the characteristics studied at ten-year intervals. The values of increases per decade, directions of trends and dynamics of changes were evaluated for each characteristic. The level of significance was set at α = 0.05. All the calculations were performed by means of STATISTICA software^TM^ (v. 12.0, StatSoft, USA).

## Results

The number of students, their age and year of examinations conducted in the period of 50 years and results of the measurements (mean±SD) of body height, body weight, BMI and body fat percentage are presented in Table 1 for both groups of students from Warsaw University of Technology (WUT) and the University of Physical Education (UPE) for compatible years of study that represented the decade-based data. The groups did not differ significantly in terms of age.

**Table 1:** Number, age, and secular trend in body height, body weight, body mass index (BMI) and fat percentage in students from the Warsaw University of Technology (WUT) and the University of Physical Education in Warsaw (UPE) in the period of 50 years.

Variables	Groups	Measurement 1	Measurement 2	Measurement 3	Measurement 4
Year of examination	WUT	1959	1971	1994	2011
	UPE	1963	1972	1996	2012
Number of students [–]	WUT	104	166	165	154
	UPE	117	120	129	107
Age [years]	WUT	19.40±1.00	21.09±1.10	20.60±0.97	20.08±0.90
	UPE	19.88±1.58	20.56±1.66	21.54±1.16	20.97±0.90
Body height [cm]	WUT	172.8±6.4*	175.1±6.1*	179.4±6.2^a^^b^	180.9±7.2^a^^b^^c^
	UPE	174.8±6.6	178.2±6.7^a^	179.8±7.1^a^	181.9±5.8^a^^b^
Body weight [kg]	WUT	64.6±7.5*	67.8±7.2^a^*	72.1± 9.0^a^^b^*	75.6±10.9^a^^b^^c^
	UPE	69.5±6.7	73.8±7.6^a^	75.2±9.2^a^	77.5±9.3^a^^b^
BMI [kg/m^2^]	WUT	21.6±1.8*	22.1±1.9*	22.4±2.5*	23.1±2.9^a^^b^
	UPE	22.7±1.7	23.2±1.7	23.2±2.1	23.4±2.1
FAT [%]	WUT	12.1±2.7*	14.2±2.7^a^*	16.2±3.0^a^^b^*	18.4±2.9^a^^b^^c^*
	UPE	12.9±1.4	12.1±2.6	15.0±2.8^a^^b^	16.4±2.6^a^^b^^c^

*Note*.^.^

^a^–means differ significantly compared to Measurement 1

^b^–means differ significantly compared to Measurement 2

^c^–means differ significantly compared to Measurement 3

p<0.05 Means WUT differ significantly compared to UPE

*—p< 0.05.

**Fig 1:**
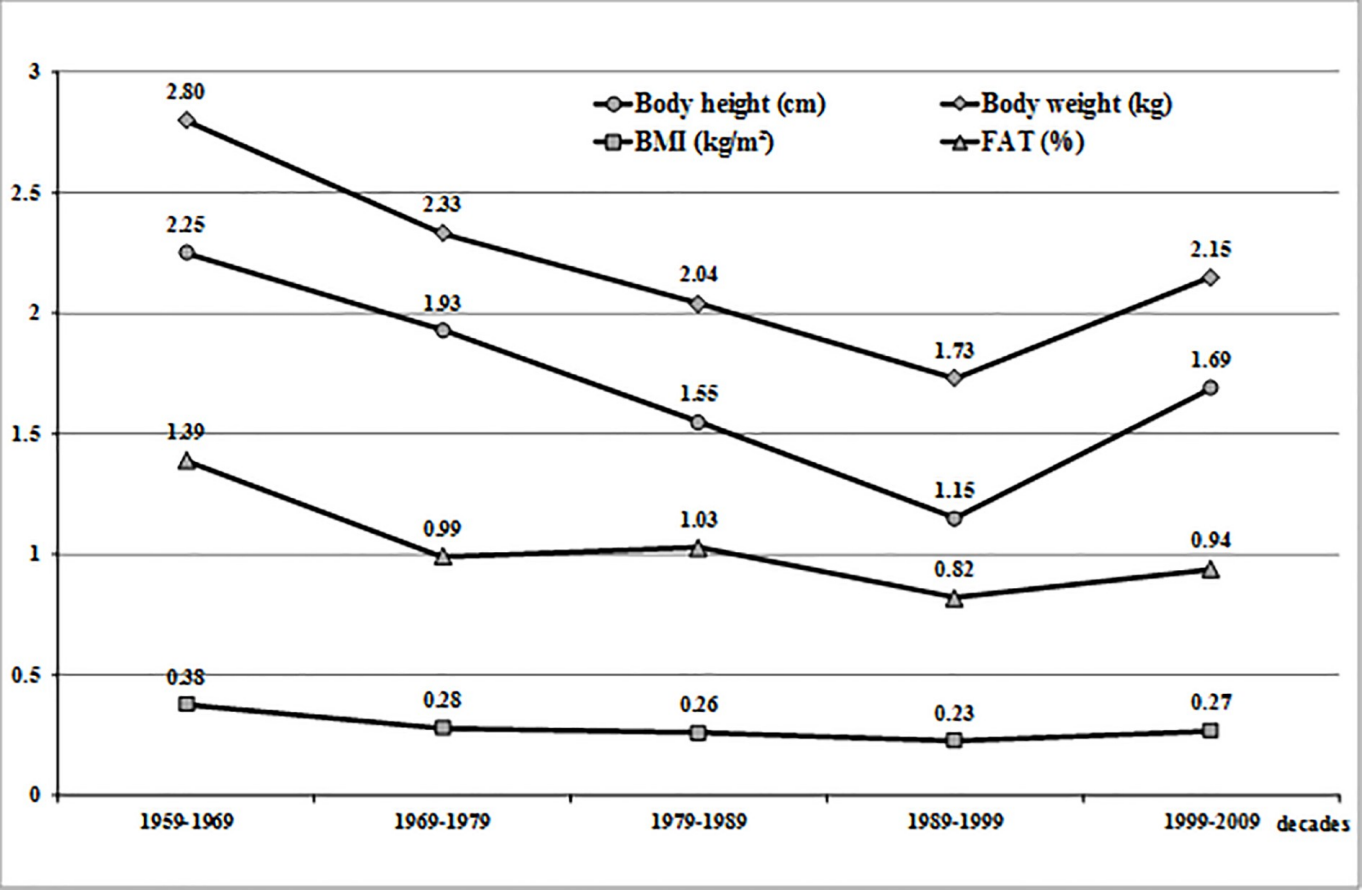
Secular trend in the studied body characteristics of students from Warsaw University of Technology from 1959 to 2009. The increases between decades were calculated from the forecast mean characteristics for 5 complete decades.

**Fig 2:**
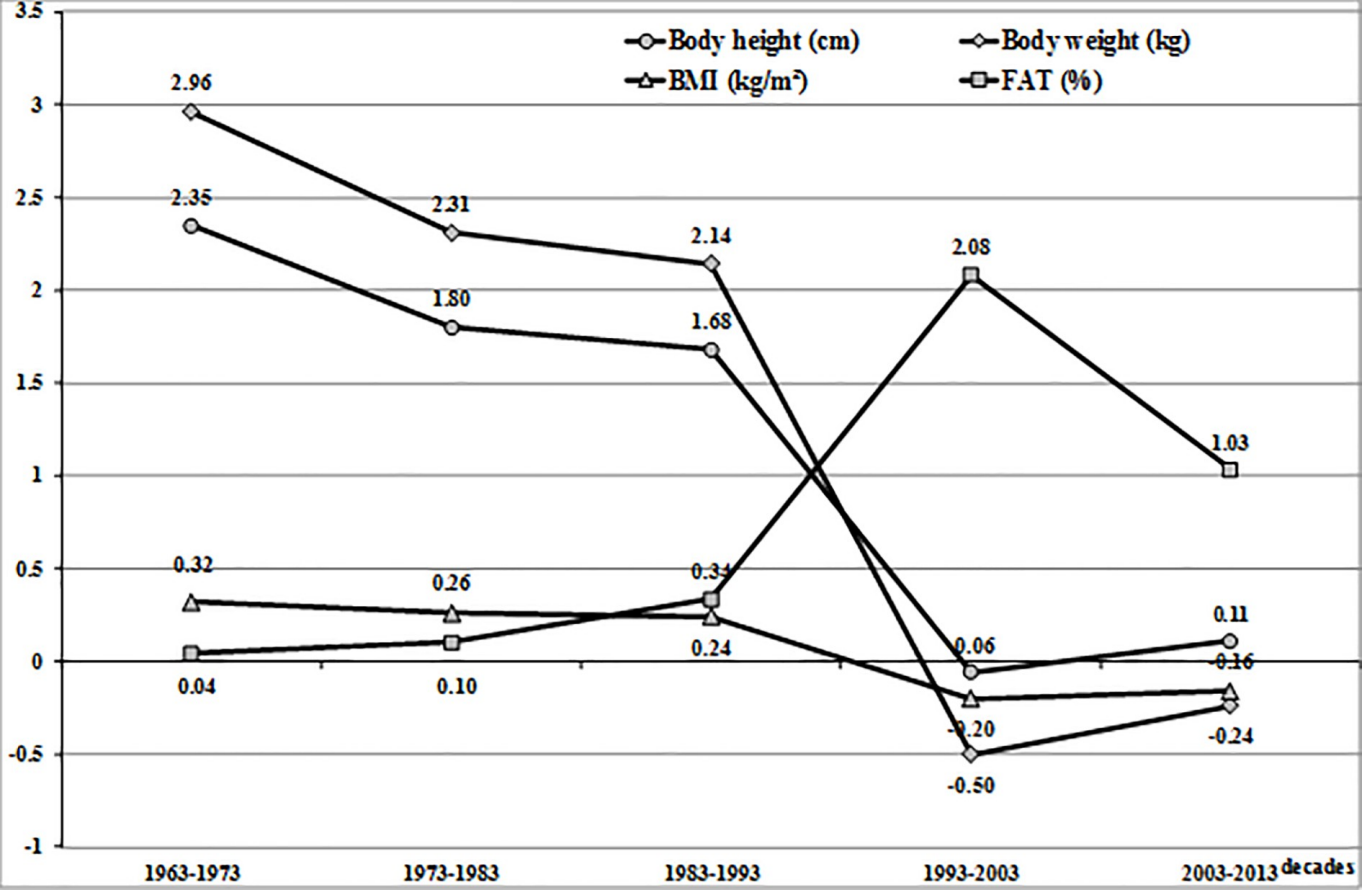
Secular trend in the studied characteristics of students from the University of Physical Education in Warsaw from 1963 to 2013. The increases between decades were calculated from the forecast mean characteristics for 5 complete decades.

The magnitude of increases per decade for each characteristic, calculated based on the means forecast for full decades, is presented in the Fig 1 for WUT students (1959–2009), whereas Fig 2 illustrates the respective data for the students from UPE (1963–2013).

Mean body height of students from WUT increased between 1959 and 2011 significantly by 8.1 cm (F_1,3_ = 44.3, p < 0.001, η^2^ = 0.1852). Post hoc Scheffé test revealed highly statistically significant differences in body height for most of the student groups, except for the years 1959/1971, 1994/2011 (Table 1). Over the whole 52-year period of observation, the increases in body height were decreasing decade by decade, which reflects the noticeable slowdown in the body height trend (Fig 1).

In the first decade (1959–1969), the difference in mean body height was 2.25 cm, whereas in the last decade (1999–2009), this difference was only 1.69 cm. The lowest trend in body height (1.15 cm per decade) was observed in 1989–1999.

The same direction of changes was found for the trend in body weight of the students from WUT. Their mean body weight in 2011 was statistically significantly higher than in the previous years of the examinations (Table 1). In 1959–2011, mean body weight increased significantly by 11.0 kg, from 64.6 kg in 1959 to 75.6 kg in 2011 (F_1.3_ = 39.94, p < 0.001, η^2^ = 0.1686). The post hoc Scheffé test revealed highly significant differences between the cohorts. The largest increase in body weight (2.8 kg) was documented in the first decade of the examinations (1959–1969), whereas the lowest body weight increase (1.73 kg), similar to body height, was observed in 1989–1999 (Fig 1). In the last decade (1999–2009), mean increase in body weight rose again to 2.15 kg.

Changes in the increases of body height and body weight in the period of the study had an effect on BMI, with its mean values increasing significantly in successive years of the examinations (F_1,3_ = 9.38, p < 0.001, η^2^ = 0.0459). BMI in the period of the analysis (1959–2011) rose from 21.6 kg/m^2^ to 23.1 kg/m^2^. The post hoc Scheffe test showed that the differences in BMI were statistically significant only in 1959/2011 and 1971/2011. The nearly linear character of BMI should be emphasized, with the biggest increase observed in 1959–1969 (0.38 kg/m^2^), followed by similar increases in the next four decades, although slightly lower (0.26 kg/m^2^ per decade, see Fig 1). Not statistically significant reduction in mean BMI to 0.23 kg/m^2^ was found in 1989–1999, and its value increased again in the last decade of the study (to 0.27 kg/m^2^).

The last analysed characteristic in the students from WUT was fat percentage. In the period of fifty two years, mean body fat percentage increased by 6.3% in the students from WUT: from 12.1% in 1959 to 18.4% in 2011 (F_1,3_ = 116.56, p < 0.001, η^2^ = 0.3736). The post hoc test demonstrated highly significant differences between the cohorts (p < 0.05). The trend line for fat percentage was smoother than trend lines for body height and body mass, whereas the increases per decade were more steady (Fig 1). The largest mean increase in fat percentage was found in the first decade of the study (1.39%) and the smallest in the fourth decade (0.82%), whereas in the last decade this value rose again by 0.94%.

A slightly different secular trend was observed for the somatic characteristics of university students from the University of Physical Education (UPE) analysed in 1963–2012 (Table 1). Mean body height of students from UPE increased significantly in the period of 49 years by 7.1 cm (F_1,3_ = 23.20, p < 0.001, η^2^ = 0.1293). The post hoc Scheffé test demonstrated highly significant differences for many of the cohorts, except for the years 1972/1996, 1996/2012. The largest mean increase in body height (2.35 cm) was observed in the first decade (1963–1973), and, in the next two decades, the increases in body height were smaller and smaller, with 1.80 cm in 1973–1983 and 1.68 cm in 1983–1993 (Fig 2).

Minimal increases in body height of ca. 0.1 cm per decade were observed over the last twenty years, suggesting a substantial slowdown of the trend in body height in this group of students.

Analogous changes were observed for the trend in body weight of the students of UPE. In 1963–2012, mean body weight of university students increased significantly by 8.0 kg (F_1,3_ = 19.03, p < 0.001, η^2^ = 0.1085). The post hoc Scheffé test demonstrated highly statistically significant differences for the cohorts studied, except for the years 1972/1996, 1996/2012.

For three next decades (1963–1993), mean increases of body weight were steadily decreasing from 2.96 kg in the first decade (1963–1973) to 2.14 kg in the third decade (1983–1993, Fig 2). In 1993–2003, the trend direction was reversed for the first time and the mean mass increases were reduced by 0.5 kg. The negative trend in body weight was also maintained in the last decade (2003–2013) at the level of -0.24 kg.

In this 49-year period of study, BMI in UPE students also changed. BMI rose from 22.7 kg/m^2^ in 1963 to 23.4 kg/m^2^ in 2012, i.e. by 0.7 kg/m^2^ (F_1,3_ = 2.53, p = 0.057, η^2^ = 0.0159). The shape of the trend line for BMI in the period studied was more stable compared to the rapidly changing pattern observed for body height and body mass (Fig 2). A substantial increase in BMI was observed in the first decade (0.32 kg/m^2^), followed by smaller decreases in the next two decades and a weak negative trend (around -0.20 kg/m^2^) in the last twenty years of the study.

In the period studied, mean body percentage in the students from UPE changed significantly. Fat percentage increased by 3.5% from 12.9% in 1963 to 16.4% in 2012. Changes in mean fat percentage in consecutive years were statistically significant (F_1,3_ = 72.94, p < 0.001, η^2^ = 0.3181), whereas the post hoc Scheffé test revealed highly significant differences for most cohorts, apart from the cohorts from years 1963/1972. Contrary to the trends in body height, body mass and BMI, the curve obtained for the increases in fat percentage was entirely atypical in this group of students (Fig 2). A negative trend in this characteristic was observed in the first decade, whereas it was steadily rising from the second to the last decade in the students from UPE. The largest increases in fat percentage were found in the two last decades: 2.08% (1993–2003) and 1.03% (2003–2013). The increases in body fat percentage (FAT%) presented in Fig 2 were forecast until 2013. Therefore, the total value of the increases in this characteristic over the whole period studied (1963–2013) is slightly greater than the actual increase in this characteristic obtained in 1963–2012.

Comparison of the results obtained between the groups of athletes (UPE) and non-athletes (WUT) revealed significant differences in body height between universities only for the 1960s and 1970s. In the case of body mass and BMI, significant differences between universities were observed in the 1960s, 1970s and 1980s, whereas no significant differences were found in the last examinations (Measurement 4). Significant differences in FAT% were observed in all the analysed years. It is apparent that after a noticeable increase in fat percentage in the group of athletes (UPE) in 1972–1996, further increases in body fat were weaker and not statistically significant, contrary to the group of non-athletes (WUT), who showed a steadily rising fat percentage in subsequent years of examinations.

## Discussion

The aim of the study was to determine changes in the magnitude and direction of secular trends in body height, body mass, BMI and fat percentage in university students from a university of technology and a university of physical education in a period of 50 years.

The major finding of the study is that in the period of the last 50 years, both groups of students showed a substantial increase in body dimensions, especially body height and body mass, and body percentage. Body height of the students from WUT increased by 8.06 cm (1.55 cm per decade), whereas in the students from UPE, this value rose by 6.90 cm (1.38 cm per decade). Students from UPE were taller than those from WUT in each examination, although the differences between these groups were gradually decreasing over the years. Similar changes in body height (7.8 cm) in 19-year-old Polish military recruits were documented by Kołodziej et al. [7] in an analogous period (1965–2010). According to these authors, the results reflected cultural changes and improvement in the financial status of the population, which contributed to more efficient utilization of the genetically determined growth potential.

The rate and magnitude of secular trends of the analysed somatic characteristics recorded during consecutive examinations of students were connected with the economic situation in Poland. The most substantial increases in most of the characteristics studied were found in the first two decades of the study. Students examined in the 1950s and 1960s were born during World War II and grew up in the very difficult conditions of the war and post-war periods. Therefore, mean dimensions of their bodies were smaller compared to the analogous dimensions of the students born later, regardless of the type of university. The nineteen seventies in Poland were a period of relative well-being, which was caused by loans financed by western banks. In this period, with the increase in living standards of average Polish families, children grew up in a more comfortable social and economic environment. A noticeable trend in body height of young people and acceleration of adolescence in Polish girls was observed in this period [13]. The 1980s recession was the worst time for the growth of children and young people. The study by Hulanicka et al. [14] demonstrated that the period of the economic recession in the nineteen eighties led to the inhibition of the trend in body height of children and young people. The financial crisis was particularly difficult for Poles, who had to cope with rationing of basic food products (butter, sugar, meat) and beginnings of tough reforms that gave rise to profound economic and systematic transformations in Poland. One of the problems was the very high costs of these transitions, reflected by the substantial regression in production, an unprecedented increase in mass unemployment, and a decline in real incomes in the major part of society.

These changes were also reflected in the dynamics of somatic growth in the groups of students. The 1980s recession influenced the trend in body height in both groups of students, leading to the smallest increases of these characteristics over the whole period of the study: 1.15 cm in the students from WUT (1989–1999) and 0.06 cm in the students from UPE (1993–2003). In a study published by Kołodziej et al. [7], the smallest increases in body height among Polish military recruits (0.80 cm) were documented in 1995–2005.

The systematic and economic transformations at the turn of the 21st century in Poland led to a substantial increase in the trend in body height, acceleration of girls' adolescence, improved level of parents' education and substantial improvement in the number of durable-use goods owned by families [13]. One of the positive effects of the transformations observed in the last decade of the study was a noticeable trend in body height in the group of students from WUT (1.69 cm) and a positive yet very weak increase in body height in the group of students from UPE (0.11 cm). A noticeable revival of the trend in body height among Polish military recruits (to 1.0 cm) in the last decade of the study was also documented by Kołodziej et al. [7].

Reports on weakening or even fading trends in body height in certain European populations, including Poland, are becoming more and more frequent. A negative trend in body height was observed in students from the Maritime University of Szczecin, examined in 1969–2007 [15]. Mean body height obtained by students from this university in 2007 was 0.16 cm lower than mean body height of students from 1990. The trend in body height in European populations was very varied. Larnkjær et al. [16] examined the trend in body height among military recruits from selected European countries who were doing obligatory military service. In Belgium, Spain and Portugal, the trend in body height of military recruits in 1980–1997 was increasing by around 2 to 3 cm per decade. In Italy, a similar rate of increase (2 cm per decade) was found only in 1960–1990, and, in the next period, from 1990 to 1997, it was only 0.5 cm. Only in the Scandinavian countries and the Netherlands was no trend in body height observed in the same period, except for military recruits from Sweden and Denmark, with very small increases in body height (0.5 cm per decade). The study by Schönbeck et al. [17] showed that the trend in body height in the Dutch stopped, with this nationality considered to be the tallest people in the world. In 2009, body height of 21-year-old Dutch people was the same as in 1997 (183.8 cm). The reasons for this trend stopping were not fully explained. It is unknown whether the trend in body height has ended and whether the lack of increases in this characteristic in subsequent years of observation is likely to suggest the full utilization of the genetic potential of the population. The last decade showed a noticeable improvement in indices of living standards of the Dutch and a reduction in differences between social and economic classes. However, in their case, these factors did not modify the trend in body height, although their strong stimulating effect on this phenomenon has often been emphasized [18–19].

Over the last 60 years, substantial changes in body height have also been observed in Croatia [6]. In 1951–2010, body height of 19-year-old boys rose by 12.2 cm whereas their mass rose by 17.3 kg. The secular trend in body size over the period studied reflected positive economic tendencies, which were stopped by war (1991–1995). The war had profound effects, both short-term, such as a reduction in body mass and BMI, and long-term, in the form of reduction in body dimensions of children born in the period of war.

In the studies on secular trends, genetic factors are considered as secondary. They only determine the threshold of individual abilities, whereas the environmental factors decide whether and how this threshold is achieved. According to Pheasant [20], the lack of a secular trend in highly industrialized countries can be explained in two manners: firstly, all children reached their own threshold of genetic potential; secondly, a certain percentage of children grow below their optimum environmental conditions; therefore, body height does not change from generation to generation any more. It is claimed that secular trends result from civilization changes, a substantial improvement in the financial standing of the population and better health care [15] [18–19].

The trend in body height is a positive phenomenon, whereas the increasingly stronger trend in body mass seems to be worrying and raises a lot of concerns all over the world. Body mass is characterized by greater lability, and it is less genetically determined than body height. Therefore, body mass can change much faster depending on the changes in the lifestyles, physical activity, nutrition, illness, etc. [21]. A positive trend in body mass was found in a study by Petranowić et al. [6], Kołodziej et al. [7], and Rębacz-Maron [15].

In the whole analysed period of the study, mean body mass of the students from UPE was higher than the mean body mass of the students from WUT. Higher body mass continued to be observed in the students from UPE despite much greater increases in this characteristic in students from WUT (2.2 kg per decade) compared to those from UPE (1.6 kg per decade). The crisis in the nineteen eighties caused a weakening trend in body mass of the students from WUT in 1989/1999, whereas another decade of substantial economic and systematic transitions substantially strengthened this trend. A much more pronounced response to the crisis was observed in students from UPE (1993–2003), with rapid reduction in mean body mass and a reversed trend, whereas the continued negative trend in body mass was observed in the period of transformation (2003–2013). The curricula in UPE impose greater physical activity on students, with 6 hours of compulsory physical education classes a week, whereas the students from WUT have only 2 hours of physical education a week. The effect of physical activity on the absolute and relative body mass and change in the tissue composition has been demonstrated by numerous studies [22–24]. It is likely that accumulation of both factors, i.e. the 1980s recession and greater physical activity in this group of students, might have had an effect on the reduction in their mean body mass, consequently reversing the trend.

BMI has most often been used to evaluate body mass due to its high correlation with body fat percentage and has often been evaluated in studies on the problems of obesity, which is very dangerous for children and young people [25]. It was found that BMI is linked to mortality risk in the general adult population. Death risk is increased in people who have low or high BMI. The trend in BMI in both groups of students was affected by the trends in body height and body mass. Despite the upward tendencies, mean BMI in the students from WUT was maintained over the whole period of observation within the reference range of the WHO classification [26], whereas the profile of this trend changed similarly to the curves for other characteristics, thus reflecting the effect of the difficult economic situation in Poland. In recent years, the mean value of BMI in the students from WUT was 23.08 kg/m^2^, which was slightly lower than the mean BMI of the students from UPE (23.39 kg/m^2^). Both means were within the normal range (18.50–24.99 kg/m^2^). In the students from UPE, the trend in BMI regularly increased for the first three decades, whereas in the two last decades, its value was decreasing, which consequently led to changes in the direction of this trend. Currently, Polish university students do not show substantial problems with obesity and overweight [21], but the opposite phenomenon is often observed, i.e. underweight [27].

The most worrying and undesirable tendency for changes in somatic characteristics of students from both universities was found in body fat percentage. The students from WUT were characterized by a significantly higher fat percentage compared to their peers from UPE. However, although body fat percentage in the students from WUT was increasing relatively steadily (1% per decade), such large increases in fat percentage in UPE were observed only in the two last decades. The increasing trend in body fat percentage observed in the students from UPE was different than the trends in other characteristics. The biggest increase in this characteristic was observed in the phase of a substantial effect of the 1980s recession (1993/2003), and further increase in body fat percentage was observed in the period of systematic and economic transformation (maintained at a high level of 1%).

Prevalence of obesity and overweight observed in increasingly younger age groups is consistent with the studies by Johnson et al. [1], Sun et al. [2], and Nagel et al. [3]. These tendencies represent a pessimistic predictor of the increasing prevalence of obesity-related diseases, such as arterial hypertension, glucose intolerance or dyslipidaemia, in future generations, even in teenagers [2]. New unhealthy tendencies for the increasing fat percentage in people with normal body weight have also emerged, observed both in children [3] and adult populations [28]. The unfavourable tendencies for the increase in body fat percentage result from reduced physical activity, sedentary lifestyles and unhealthy eating habits, including consumption of high-calorie foods with low quality, increase in the amount of food consumed and growing popularity of fast food [29].

In conclusion, the changes in the characteristics analysed in this study, which represent the biological measures of living standards, show that the period of systematic and economic transformations was more beneficial for physical growth of university students than the previous period of the financial crisis. Nowadays, Polish university students are taller and heavier, characterized by normal (average) BMI, whereas some unfavourable changes in their body build are being observed, reflected by the increasing body fat percentage.

## Supporting information

S1 Data Data to calculation. (XLSX)
